# Multi-centric evaluation of Truenat MTB and MTB-RIF Dx assays for diagnosis of extrapulmonary tuberculosis

**DOI:** 10.1038/s41598-024-64688-z

**Published:** 2024-07-08

**Authors:** Urvashi B. Singh, Manjula Singh, Camilla Rodrigues, D. J. Christopher, Neeraj Mahajan, Abhinav Srivastav, Kh Jitenkumar Singh, Sunita Kanswal, M. V. V. Rao, Mubin kazi, Damini Sawant, Balamugesh Thangakunam, Coelho Victor Vijay, Deepa Shankar, Anuj Bhatnagar, Anant Mohan, Vineet Ahuja

**Affiliations:** 1 Department of Microbiology, AIIMS, New Delhi, India; 2 Division of Epidemiology and Communicable Diseases, ICMR, New Delhi, India; 3 Department of Microbiology, P D Hinduja Hospital, Mumbai, India; 4 Department of Pulmonary Medicine, CMC, Vellore, India; 5 National Institute of Medical Statistics, ICMR, New Delhi, India; 6 Centralized Core Research Facility, AIIMS, New Delhi, India; 7 Department of Tuberculosis and Chest Diseases, Rajan Babu Institute for Pulmonary Medicine and Tuberculosis, New Delhi, India; 8 Department of Pulmonary Medicine, AIIMS, New Delhi, India; 9 Department of Gastroenterology, AIIMS, New Delhi, India

**Keywords:** Extrapulmonary tuberculosis, Truenat MTB-RIF, GeneXpert MTB/RIF, Composite reference standards, Microbiological reference standards, Microbiology, Molecular biology

## Abstract

Extra-pulmonary TB (EPTB) is difficult to diagnose due to paucibacillary nature of disease. Current study evaluated accuracy of Truenat MTB and MTB-Rif Dx (TN), for detection of *Mycobacterium tuberculosis* and resistance to rifampicin. Samples were collected from 2103 treatment naive adults with presumptive EPTB, and tested by smear microscopy, liquid culture (LC) (MGIT-960) and GeneXpert MTB/RIF (GX) (Microbiological Reference Standards, MRS). TN results were compared to MRS and Composite Reference Standards (CRS, Microbiology, histopathology, radiology, clinical features prompting decision to treat, response to treatment). CRS grouped patients into 551 confirmed, 1096 unconfirmed, and 409 as unlikely TB. TN sensitivity and specificity was 73.7% and 90.4% against GX. Against LC, Overall sensitivity of GX was 67.6%, while that of TN was 62.3%. Highest sensitivity by TN was observed in pus samples (89%) and highest specificity (92%) in CSF samples, similar to GX. TN sensitivity was better in fluid and biopsy samples and slightly inferior for lymph node aspirates compared to GX. TN sensitivity for RIF resistance detection was slightly superior to GX. TN and GX results were further compared to Clinical Reference Standards. TN detected 170 TB patients initiated on treatment missed by GX, while GX detected 113 such patients missed by TN. Of 124 samples with RIF resistance discordance between GX and TN, GX reported 103/124 as sensitive, 3/124 as indeterminate and 18 as resistant (13/18 samples had low/very low DNA load) while TN reported RIF resistance indeterminate in 103/111 low/very low DNA load samples. Due to paucibacillary nature of EPTB samples, culture yield was poor and phenotypic drug susceptibility testing failed to resolve the discordance. The study establishes TN *at par* with GX and can be utilized for quick and accurate diagnosis of EPTB.

## Introduction

Tuberculosis (TB) continues to contribute to high morbidity and mortality worldwide. As per WHO estimates, it accounted for approximately 10 million new cases and 1.5 million deaths in 2020^1^. Pulmonary TB remains the primary target for TB control as it accounts for approximately 85% of all TB cases, while extra-pulmonary tuberculosis (EPTB) accounts for about 8% to 24%^2^. Annual global incidence of EPTB has been increasing over the last decade due to the changing TB control practices, the spread of human immunodeficiency virus (HIV), population growth, and improved detection. In patients with TB and HIV co**-**infection, the rate of EPTB is reported to be more than 50% of all cases^3^.

In resource-limited settings, EPTB poses an extensive diagnostic and therapeutic challenge, due to its paucibacillary nature. It is difficult to diagnose with routine diagnostic methods such as culture and acid-fast bacilli (AFB) staining. Furthermore, EPTB has complex clinical presentation, depending on the site and stage of infection and often needs invasive procedure to obtain appropriate specimens, making diagnosis more challenging. Routine cytology and histology cannot differentiate between the diseases caused by TB or non-tubercular mycobacteria, chronic inflammatory conditions, and some other granulomatous conditions. EPTB is thus diagnosed by combination of various diagnostic modalities^4^.

Early diagnosis and detection of drug resistance is crucial for effective treatment and prevention of spread of severe forms of disease in the community. With the advent of Nucleic Acid Amplification tests (NAAT), such as GeneXpert/MTB/RIF and Xpert/RIF/Ultra, the sensitivity of detection of EPTB has improved. MolBio Diagnostics (Goa, India) developed a few NAAT assays (Truenat MTB, Truenat MTB Plus and Truenat MTB-RIF Dx) based on chip-based real-time Polymerase Chain Reaction (PCR) technology that utilizes *nrdB* gene (Truenat MTB), *nrdz* and *IS6110* gene (Truenat MTB Plus) for the semi-quantitative detection of *Mycobacterium tuberculosis* (MTB) and *rpoB* gene (Truenat MTB-RIF Dx) for the detection of rifampicin resistance.

The present study was designed as a blinded, cross sectional evaluation study, to evaluate the diagnostic performance of Truenat MTB and MTB-RIF Dx assays (TN) in comparison to the Microbiological reference standards (MRS) (WHO recommended diagnostics (WRD): smear microscopy (Ziehl Neelsen stain), liquid culture (LC) (MGIT-960) and GeneXpert/MTB/RIF) (GX) (molecular WRD/mWRD) and Composite reference standards (CRS) (microbiological investigations, histopathology, radiology, clinical features prompting decision to treat for TB, response to treatment) in patients with presumptive EPTB.

## Material & methods

A multicenter, blinded, prospective study was conducted at three tertiary care centers i.e., All India Institute of Medical Sciences New Delhi, Christian Medical College, Vellore, and Hinduja National Hospital, Mumbai from August 2018 to August 2019. Consecutive patients above 18 years of age with unexplained fever, lethargy, weight loss or failure to thrive with specific extra-pulmonary presentations, such as lymph node enlargement, abscess, features of meningitis, pleural effusion, ascites and symptoms suggestive of presumptive EPTB, were enrolled after obtaining written, informed consent.

Patients with poor general condition, with high chance of mortality, Immuno-compromised state except HIV infection, co-morbid conditions like malignancy, congenital defects, those who had previously received anti-tubercular drugs were excluded.

### Sample collection and processing

Specimens including cerebrospinal fluid (CSF), peritoneal/pleural fluid, lymph node, biopsy sample and pus were collected as appropriate. Samples such as lymph-node aspirates, biopsy and pus were subjected to standard N-acetyl-L-cysteine sodium hydroxide (NALC-NaOH) decontamination and concentration by centrifugation, while samples from sterile sites were processed directly. Samples were aliquoted into two vials and assigned code numbers (generated daily by statistician, KJS). The laboratory was blinded to the clinical presentation and supporting findings of the patients. The processed samples were subjected simultaneously to AFB smear, GX, LC and TN. All conventional procedures for smear, LC and GX were performed following standard recommendations^5–7^. TN results were recorded against the respective code number assigned.

### Acid- Fast *Bacillus* (AFB) smear microscopy

Ziehl–Neelsen (ZN) staining was performed on both direct and decontaminated samples. Smears were examined under light microscope for the presence of acid-fast bacilli^5,6^.

### Liquid culture and liquid culture drug susceptibility testing (DST)

The processed samples were inoculated into MGIT 960 (Becton Dickinson, MD, USA). The tubes were incubated at 37 °C and the ones flagged positive were tested for contamination on Mueller Hilton Agar plate followed by confirmation by smear microscopy and TBc identification test (Becton Dickinson, MD, USA). Drug susceptibility testing for rifampicin (RIF) and isoniazid (INH) was performed with the MGIT 960 system, using WHO recommended standard critical concentration of 1 µg/ml for RIF and 0.1 µg/ml for INH.

### GeneXpert MTB/RIF (GX)

The GX assay was performed according to manufacturer’s instructions (Cepheid, Sunnyvale, CA). Sample reagent was added in a 2:1 ratio to unprocessed sterile sample or decontaminated sample collected from non-sterile sites, and the tube was manually agitated twice during a 15 min incubation period at room temperature. From the treated sample 2 ml was transferred into the cartridge and loaded onto the GeneXpert instrument. The GeneXpert Dx software (version 4.0, Cepheid) reports results as Mycobacterium complex (MTBC) detected, not detected, invalid, error or no result. The resistance to RIF was reported as RIF resistance detected, not detected or indeterminate. The indeterminate result was reported when the test could not accurately determine whether the bacteria were resistant to RIF. The test was repeated for invalid, error, no result, or indeterminate results for RIF resistance^7^.

### Truenat MTB and Truenat MTB-RIF (TN)

Truenat MTB and Truenat MTB-RIF were performed as per manufacturer’s instructions (MolBio Diagnostics, Goa, India). The samples were processed using the Trueprep AUTO sample pre-treatment pack and the DNA was extracted adding two drops of liquefaction buffer to 0.5 ml of sample. The entire content was then transferred to lysis buffer tube and incubated for 5 min. This mixture was then transferred into the cartridge and loaded on to the device to isolate ~ 40 µl of DNA. For amplification 6 µl of purified DNA was added to microtube containing lyophilized master-mix and allowed to stand for 30–60 s to get a clear solution. A 6 µl of this clear solution was dispensed into the reaction well of the Truenat MTB chip and loaded to the Truenat Uno Dx instrument. The results were displayed as amplification curve on the analyzer screen on a real time basis during the test run. At the end of the test run the results were reported as MTB detected, not detected or invalid. The test was repeated for invalid results. Indeterminate or error was displayed when the obtained values did not meet the requirements for resistance determination. The test was repeated for sample displaying indeterminate or error^8^.

All experiments were performed in accordance with the National ethical guidelines for biomedical and research health involving human participants, 2017 (available at:

https://ethics.ncdirindia.org/ICMR_Ethical_Guidelines.aspx).

Microbiological Reference Standards (MRS) included LC, Smear and GX^8,9^. Results of TN were compared to MRS, Smear or Liquid Culture results taken together and to liquid culture alone as well. In order to compare the performance of the two molecular tests, similar comparison was done for GX.

Composite Reference Standards (CRS) included microbiological investigations, histopathology, radiology, clinical features (weight loss, fever, lymph node, night sweats, history of contact and loss of appetite) prompting decision to treat for TB, response to treatment. Patients were grouped as Confirmed/Unconfirmed and Unlikely TB^4,10^.

### Statistical analysis

Data were analyzed using Stata version 14.1 (StataCorp, College Station, TX). Performance of TN was determined statistically in comparison with MRS and CRS using STATA. Performance of TN and GX were compared with LC and with Clinical Reference Standards in addition, using STATA. Data analysis was done per patient.

### Ethics

Institute Ethics Committee (IEC), All India Institute of Medical Sciences, New Delhi approved the study protocol (IEC No. IEC-5/09.02.2017). TB treatment decisions were not made based on the result of the TN assay under evaluation, but based on the clinical work-up along with MRS.

## Results

The three sites namely All India Institute of Medical Sciences New Delhi, Christian Medical College, Vellore, and Hinduja National Hospital, Mumbai contributed 843, 348 & 912 samples respectively. There were 1241 (59%) samples from male patients and remaining 862 (41%) from female patients.

Five types of samples were included in the study, 202 (9.61%) were CSF, 644 (30.62%) were fluids (peritoneal, pleural), 449 (21.35%) were lymph node aspirates, 404 (19.21%) were pus and tissue biopsy each respectively. Of all, 19.6% patients presented with cough, 36% with fever, 25.34% had weight loss in recent months, 7.4% patients had abdominal pain, 6.4% had headache and 21.63% had lymphadenopathy.

Of 2103 samples analyzed 358 samples (17%) were found positive using MGIT culture test, 429 samples (20.4%) were found positive using GX and 486 (23.11%) were positive by TN (Table 1).

**Table 1:** Comparative analysis of test results by TN with WRD.

Smear (ZN)							Culture (MGIT)						m WRD (GX)				
TN		Pos	Neg	Total	Sensitivity 89·2 (80·4, 94·9) Specificity 79·6 (77·8, 81·3) PPV 15·2 (12·1, 18·7) NPV 99·4 (98·9, 99·7)	TN		Pos	Neg	Total	Sensitivity 62·3 (57, 67·3) Specificity 84·9 (83·2, 86·6) PPV 45·9 (41·4, 50·4) NPV 91·7 (90·2, 93)	TN		Pos	Neg	Total	Sensitivity 73·7 (69·2, 77·8) Specificity 89·8 (88·3, 91·3) PPV 65 (60·6, 69·3) NPV 93 (91·7, 94·2)
	Pos	74	412	486			Pos	223	263	486			Pos	316	170	486	
	Neg	9	1608	1617			Neg	135	1482	1617			Neg	113	1504	1617	
	Total	83	2020	2103			Total	358	1745	2103			Total	429	1674	2103	

### Comparison of TN with AFB smear

TN test gave a sensitivity of 89.2% in comparison with smear results and specificity of 79.6%. The positive predictive value of TN was 15.2% and negative predictive value was 99.4% (Table 1).

### Comparison of TN with gold standard LC for detection of TB

TN when compared with LC as gold standard for EPTB diagnosis gave 62.3% sensitivity and 84.9% specificity (Table 1). The sensitivity, specificity of GX was 67.6% and 89.3% in all sample types collected (Table 3).

The sensitivity of TN compared to LC in AFB positive specimens was 93.2% while that of GX was 96.6%. Sensitivity in AFB negative specimens was 56.2% while GX had a sensitivity of 61.9% (Table 2).

**Table 2:** Diagnostic performance of TN & mWRD in smear positive and smear negative specimens with MGIT culture.

AFB smear positive (n = 83)	TN	MGIT			MGIT			
		Pos	Neg	Total	GX	Pos	Neg	Total
	Pos	55	19	74	Pos	57	18	75
	Neg	4	5	9	Neg	2	6	8
	Total	59	24	83	Total	59	24	83
Sensitivity		93·2 (83·5, 98·1)			Sensitivity	96·6 (88·3, 99·6)		
Specificity		20·8 (7·13, 42·2)			Specificity	25 (9·77, 46·7)		
PPV		74·3 (62·8, 83·8)			PPV	76 (64.7, 85.1)		
NPV		55.6 (21.2, 86.3)			NPV	75 (34·9, 96·8)		

AFB smear negative (n = 2020)	Truenat	MGIT			MGIT			
		Pos	Neg	Total	GeneXpert	Pos	Neg	Total
	Pos	168	244	412	Pos	185	169	354
	Neg	131	1477	1608	Neg	114	1552	1666
		299	1721	2020		49	1721	2020
Sensitivity			56.2 (50.4, 61.9)		Sensitivity		61.9 (56.1, 67.4)	
Specificity			85.8 (84.1, 87.4)		Specificity		90.2 (88.7, 91.5)	
Positive predictive value			40.8 (36, 45.7)		Positive predictive value		52.3 (46.9, 57.6)	
Negative predictive value			91.9 (90.4, 93.1)		Negative predictive value		93.2 (91.8, 94.3)	
Positive likelihood ratio			3.96 (3.4, 4.62)		Positive likelihood ratio		6.3 (5.32, 7.46)	
Negative likelihood ratio			0.51 (0.45, 0.58)		Negative likelihood ratio		0.42 (0.36, 0.49)	

The sensitivity and specificity of TN varied with sample types collected in our study. Pus samples demonstrated highest sensitivity of 89% while lowest sensitivity of 43.8% was detected in fluid samples. Further, highest specificity was seen in CSF (92%) while lowest specificity of 69% was seen in pus samples (Table 3). Similar to results in TN, GX demonstrated highest sensitivity of 89% in pus and specificity of 91.5% in CSF. The lowest sensitivity of GX, 31.3% was seen in fluid samples (43.8% by TN) and specificity of 78.9% in pus samples (69% by TN) (Table 3).

**Table 3:** Comparative analysis of TN and mWRD (GX) with Culture (MGIT) (in all samples and sample type wise).

TN	Culture (MGIT)				GX	Culture (MGIT)			
	Pos	Neg	Total			Pos	Neg	Total	
Pos	223	263	486	Sensi: 62·3 (57, 67·3) Spec: 84.9 (83.3, 86.6)	Pos	242	187	429	Sensi: 67·6 (62·5, 72·4) Spec: 89.3 (87.7, 90.7)
Neg	135	1482	1617	PPV: 45.9 (41.4, 50.4)	Neg	116	1558	1674	PPV: 56.4 (51.6, 61.2)
Total	358	1745	2103	NPV: 91.7 (90.2, 93)		358	1745	2103	NPV: 93.1 (91.7, 94.2)
CSF									

TN	MGIT				MGIT				
	Detected	Not detected	Total		Xpert	Detected	Not detected	Total	
Detected	2	16	18	Sensi: 66·7 (9·43, 99·2) Spec: 92 (87.3, 95.3)	Detected	2	17	19	Sensi: 66·7 (9·43, 99·2) Spec: 91.5 (86.7, 94.9)
Not Detected	1	183	184	PPV: 11.1 (1.38, 34.7)	Not Detected	1	182	183	PPV: 10.5 (1.3, 33.1)
Total	3	199	202	NPV: 99·5 (97, 100)	Total	3	199	202	NPV: 99·5 (97, 100)
FLUID									

TN	MGIT				Xpert	MGIT			
	Detected	Not detected	Total			Detected	Not Detected	Total	
Detected	21	75	96	Sensi: 43·8 (29·5, 58·8) Spec: 87.4 (84.5, 90)	Detected	15	40	55	Sensi: 31·3 (18·7, 46·3) Spec: 93.3 (91, 95.2)
Not Detected	27	521	548	PPV: 21.9 (14.1, 31.5)	Not Detected	33	556	589	PPV: 27.3 (16.1, 41)
Total	48	596	644	NPV: 95.1 (92.9, 96.7)	Total	48	596	644	NPV: 94.4 (92.2, 96.1)
LYMPH NODE									

TN	MGIT				Xpert	MGIT			
	Detected	Not detected	Total			Detected	Not detected	Total	
Detected	77	48	125	Sensi: 55·4 (46·7, 63·8) Spec: 84·4 (80, 88·4)	Detected	100	42	142	Sensi: 71·9 (63·7, 79·2) Spec: 86·5 (82·1, 90·1)
Not Detected	62	262	324	PPV: 61.6 (52.5, 70.2)	Not Detected	39	268	307	PPV: 70·4 (62·2, 77·8)
Total	139	310	449	NPV: 80·9 (76·2, 85)	Total	139	310	449	NPV: 87.3 (83, 90.8)
PUS									

TN	MGIT				Xpert	MGIT			
	Detected	Not detected	Total			Detected	Not detected	Total	
Detected	81	97	178	Sensi: 89 (80·7, 94·6) Spec: 69 (63.6, 74.1)	Detected	81	69	150	Sensi: 89 (80·7, 94·6) Spec: 78 (72.9, 82.4)
Not detected	10	216	226	PPV: 45·5 (38, 53·1)	Not Detected	10	244	254	PPV: 54 (45·7, 62·2)
Total	91	313	404	NPV: 95·6 (92, 97·9)	Total	91	313	404	NPV: 96·1 (92·9, 98·1)
TISSUE BIOPSY									

TN	MGIT				Xpert	MGIT			
	Detected	Not detected	Total			Detected	Not detected	Total	
Detected	42	27	69	Sensi: 54·5 (42·8, 65·9) Spec: 91.7 (88.2, 94.5)	Detected	44	19	63	Sensi: 57·1 (45·4, 68·4) Spec: 94.2 (91.1, 96.5)
Not detected	35	300	335	PPV: 60.9 (48.4, 72.4)	Not Detected	33	308	341	PPV: 69.8 (57, 80.8)
Total	77	327	404	NPV: 89.6 (85.8, 92.6)	Total	77	327	404	NPV: 90.3 (86.7, 93.2)

### Comparison of TN with GX

TN in comparison with GX gave sensitivity of 73.7% and specificity of 89.8%, positive predictive value of 65% and negative predictive value of 93% (Table 1).

### Comparison of TN with microbiological reference standards

Comparing the results of TN with the MRS (smear, culture, GX), the sensitivity was 63% and specificity was 91%, positive predictive value of 71.4% and negative predictive value of 87.4% (Supplementary Table 1).

### Comparative performance of TN with GX for detection of RIF resistance

GX detected 360 (17.12%) samples to be sensitive, 55 (2.61%) as resistant and 13 (0.62%) as indeterminate, whereas TN found 234 (11%) sensitive, 39 (1.9%) as resistant and 206 (9.79%) as indeterminate results (Table 4). The resultant concordance between TN and GX was seen in 80.8% of specimens.

**Table 4:** Comparative analysis of TN results with mWRD (GX).

TN RIF	Sensitive	GX RIF			
		Resistant	Indeterminate	Not detected	Total
Sensitive	165	5*	2*	62	234
Resistant	5*	20	1*	13	39
Indeterminate	98*	13*	5	90	206
Not detected	92	17	5	1510	1624
Total	360	55	13	1675	2103

Concordance: 1700/2103 = 80·8%

*Discordant results observed in a total of 124 samples tested, detailed analysis of the same is provided in Supplementary table no. 2

The discordant results were seen in 124 samples, highest in lymph node (48) followed by pus (39), tissue biopsy (22), fluid (11) and CSF (4). The discordant results observed were seen primarily in samples with low or very low DNA levels in 113 (91.12% of discordant) samples (Supplementary Table 2). Rifampicin resistance by TN was indeterminate in 111/124 (89.5%) samples and 103/111 (92.8%) samples had low/very low DNA quantity. In contrast, GX gave 103/124 samples as sensitive despite 85/103 (82.5%) with low and very low amount of DNA, and 18/124 as resistant, though 13/18 (72.22%) had low/very low DNA. In 3/124, GX gave indeterminate results (Supplementary Table 2).

### Comparison of TN with composite reference standards

On further analysis of molecular test results with Composite Reference Standards, Confirmed, Unconfirmed and Unlikely TB, TN gave a sensitivity of 63% and specificity of 91.6% (Table 5). Positive predictive value for TN was 79% and Negative Predictive value of 83.1%.

**Table 5:** Concordance of Truenat with Composite Reference Standard (Confirmed, Unconfirmed and Unlikely TB).

	Confirmed TB (Clinical + Microbiological Evidence) (Xpert/MGIT/AFB positive)	Unconfirmed TB (Clinical/ Symptomatic Evidence) (Xpert/MGIT/AFB negative)	Unlikely TB (Neither microbiological nor clinical /symptomatic evidence)	Total
*Truenat*				
Detected	347	92	47	486
Not Detected	204	1004	409	1617
Total	551	1096	456	2103
Sensitivity			63 (58·8, 67)	
Specificity			91·6 (89·8, 93·2)	
Positive predictive value			79 (74·9, 82·8)	
Negative predictive value			83·1 (80·9, 85·2)	
Positive likelihood ratio			7·5 (6·11, 9·22)	
Negative likelihood ratio			0·4 (0·36, 0·45)	

### Comparison of TN and GX results with clinical reference standards (composite reference standards without microbiological reference standards)

In order to compare the performance of TN and GX, patients were grouped using clinical features, radiology, histopathology, and other accessory investigations but without microbiological investigations as Confirmed, Unconfirmed and Unlikely TB^10^. TN gave a sensitivity of 69.6% and specificity of 99.9%, while GX gave a sensitivity of 61.5% and specificity of 100% (Supplementary Table 3 (a). Positive predictive value for TN was 99.8% (GX, 100%) and Negative Predictive value of 84% (GX, 80.6%).

### Comparison of TN positive and GX negative results with clinical reference standards

TN detected 170 extra samples which were missed by GX, smear and culture. Of these patients, 169/170 were Confirmed TB, while 1 patient was Unconfirmed TB. Of these, 59 (34.9%) samples had high DNA quantity but were missed by GX (Supplementary Table 3 (b). In contrast, samples that were detected by GX but missed by TN (Supplementary Table 3 (c) primarily had low/very low quantity DNA (53.09%). This trend was seen in combined as well as site specific data.

## Discussion

Initial studies evaluating GX gave sensitivity of detection of TB in the range of 25%-95%. The differences in these studies were primarily due to enrollment of different patient populations, different clinical presentations of EPTB, different sample types, processing techniques and comparator gold standards utilized^11^. WHO recommended the use of GX in EPTB samples^12^. However, the yield of GX was low in paucibacillary samples due to limited amount of DNA detected^13^. Several meta-analyses concluded sensitivity from 69 to 83%^14–16^. With the new Xpert Ultra, based on detection of two multicopy gene sequences, improvement in sensitivity was reported^17^. Studies using Xpert Ultra on EPTB samples have shown improved performance^18–20^. Some studies with small sample size have found, diagnostic yield of 77.14% with Truplus and 59.18% with Xpert Ultra in 100 lymph node samples^21^; another study found a yield of 3.6% in lymph nodes samples. Rajendran et al. reported sensitivity of 60% with TN in 298 EPTB samples^23^.

In the current study, the sensitivity of detection by TN in comparison to LC was 62.3% while specificity was 85%, close to that of GX, 67.6% and 89.3%, similar to previous studies in EPTB samples where sensitivity and specificity of TN compared to LC was 65% and 70% respectively^24^. Initial studies evaluating GX had demonstrated similar sensitivity (79%) and specificity (97%) when compared to LC^25^.

Sensitivity for different extra-pulmonary samples varied, being highest in pus samples, similar by both TN and GX (89%) and lowest in pleural/peritoneal fluids (43.8% by TN, 31.3% by GX). TN gave similar sensitivity to GX for pus samples and CSF, higher sensitivity than GX in fluids and biopsy, and lower sensitivity than GX in lymph node aspirates. Inhibitors of PCR such as high protein content, factors released during clotting cycle, heme in samples could cause inhibition of PCR leading to different yield with different sample types. Published work has reported GX sensitivity and specificity of 71.1% and 98% in CSF, 87.6% and 86% in lymph node aspirates and 50.9% and 99% for pleural fluid^26^. In studies using GX Ultra, sensitivity from lymph nodes has been reported from 50 to 100%, for CSF 71.4 to 96.4%, and lower sensitivity for pleural fluids 47.6 to 84.2%^27^.

Further, sensitivity of RIF resistance detection by TN in comparison to LC DST was 95% while sensitivity of RIF resistance detection by GX was 94.4%. In a previous study published by our group sensitivity of RIF resistance detection was 81.82% with GX^28^. There was a concordance of 80.8% (1700/2103) in the RIF resistance results by TN and GX. Of 124 samples showing discordant results, substantial number had low/very low amount of DNA. While TN labeled 111/124 as indeterminate RIF resistance, GX reported 103/124 as sensitive, 3/124 as indeterminate and 18/124 as resistant despite the low/very low DNA. Low/very low amount of DNA is known to create confusion in detection of RIF resistance due to fallacies in probe binding in GX and failing amplification in TN. Reports caution against inference of resistance in samples with low/very low DNA. Paucibacillary nature of extra-pulmonary samples complicates detection of RIF resistance^29–32^.

With respect to Composite Reference Standards (CRS), detection by TN in patients grouped as Confirmed, Unconfirmed and Unlikely TB gave sensitivity of 63% & specificity of 91.6%. Similar studies with GX have shown a sensitivity in similar range. Collection and processing of extra-pulmonary samples offers a challenge. While GX extraction protocol involves decontamination by NaLC-NaOH method followed by centrifugation for all extra-pulmonary samples (except CSF) and loading into the cartridge for GX; TN protocol recommends addition of sample treatment buffer directly to samples (to pellet after centrifugation for CSF, pericardial/peritoneal/pleural fluids) and loading into Autoprep for TN. Paucibacillary samples could possibly have additional loss of bacterial DNA during extraction. [TN/GX kit instructions].

With respect to Clinical Reference Standards (CRS without microbiological investigations), detection by TN in patients grouped as Confirmed, Unconfirmed and Unlikely TB gave sensitivity of 69% & specificity of 99.9%. GX performed slightly inferior with a sensitivity of 61.5% and specificity of 100%. TN detected 170 extra samples (at all three sites), which were missed by GX and other microbiological tests. These patients were all in Confirmed TB group with one patient in Unconfirmed TB group. The amount of DNA in these samples was low/very low in 57% patients, and high in 35%, but was missed by GX. GX detected 113 samples missed by TN (included for TN sensitivity calculation, TN showed sensitivity of 73.7% in comparison to GX). These samples predominantly had low/very low DNA amount, and could have hence been missed by TN^28,33,34^.

Age and immune response can significantly affect the disease immunopathogenesis which could in turn affect the disease presentation and hence detection by NAAT. We reported yield of 24.1% by GX in respiratory samples from children with presumptive intra-thoracic TB, significantly higher (31%) in children with progressive primary TB (lung parenchymal lesion) and 11.9% in those with primary pulmonary complex disease (hilar lymphadenopathy)^35^. Kabir et al.^36^reported higher yield of GX in males and in young adults.

Few limitations are that the study was done in initial versions of TN and Genexpert MTB/RIF. Further improved versions are now available for both systems. Future versions of NAATs could include simplifying sample processing and inclusion of more genes in the test design to improve sensitivity of TB detection and EPTB diagnosis. Also, inclusion of more gene targets could enable detection of resistance to important drugs in the treatment regimen such as Bedaquiline, Linezolid, INH and fluoroquinolones, essential for designing treatment regimen for drug resistant TB. Both TN and GX missed few samples detected by the other test possibly due to low amount of DNA. TN gave Rif resistance indeterminate results in samples with low amount of DNA, which should be improved in future versions. GX reported a small number of low DNA samples as resistant. WHO recommends phenotypic DST or repeat sample testing in samples with lower bacillary load^37^, however repeating extra-pulmonary samples may not always be feasible and paucibacillary samples may have poor yield on culture. Newer technology or improvements in the current technology could overcome this dilemma.

Strengths of the current study include a large sample size included from three tertiary care centers in a high endemic, low resource setting. All patient samples were systematically subjected to WHO recommended diagnostics which were used as gold standard comparator for evaluation of TN. Composite reference standards including clinical suspicion, microbiological investigations, histopathology, radiology and response to treatment were used together to further evaluate test results.

In patients initiated on treatment, TN achieved sensitivity of 69.6% (65.1% for GX) and a higher negative predictive value than GX. TN proved to be instrumental in diagnosing more patients with confirmed TB, where other diagnostic methods failed. The current study establishes TN as a sensitive and specific technique for diagnosis of EPTB. TN can work on a hand-held device (battery operated), with minimal operational requirements and potential to be utilized at peripheral centres in resource limited settings. The assay involves automated DNA extraction in Trueprep device (20 min) which is loaded on a chip and amplified in Truelab micro-PCR device (40 min).GX system requires ambient temperature between 15 and 30 °C, stable electricity supply, and adequate storage space for cartridges (storage at 2–28 °C ), thereby, limiting its use to district hospital settings.

Despite advances in diagnostic technology, treatment is initiated based on comprehensive reference standards in EPTB. In paucibacillary samples, the results by both methods for samples such as fluids and biopsy as well as RIF resistance detection was imperfect, prompting scope for improvements in diagnostic modalities.

### Supplementary Information

Supplementary Tables.

## Data Availability

The datasets generated during and/or analysed during the current study are available from the corresponding author on reasonable request.
