# Supplementary material for: Multi-centric evaluation of Truenat MTB and MTB-RIF Dx assays for diagnosis of extrapulmonary tuberculosis
Source: Sci Rep. 2024 Jul 8;14:15680. doi: 10.1038/s41598-024-64688-z (PMC11231208; doi:10.1038/s41598-024-64688-z)
Supplement: Supplementary file 1 — Supplementary Tables. [file 41598_2024_64688_MOESM1_ESM.docx]

**Supplementary Tables**

**Table 1. Performance of TN with Microbiological reference standard (MRS)**

|  | **Smear (ZN) or**  **Culture (MGIT) or mWRD (GX)** | **Smear (ZN) And**  **Culture (MGIT) And mWRD (GX)** |  |
| --- | --- | --- | --- |
| **TN** | **Pos** | **Neg** | **Total** |
| **Pos** | 347 | 139 | 486 |
| **Neg** | 204 | 1413 | 1617 |
| **Total** | 551 | 1552 | 2103 |
| Sensitivity 63 (58·8, 67)  Specificity 91 (98·5, 92·4)  Positive predictive value 71·4 (67·2, 75·4)  Negative predictive value 87·4 (85·7, 89)  Positive likelihood ratio 7·03 (5·93, 8·34)  Negative likelihood ratio 0·40 (0·36, 0·45) | | | |

**Table 2. Comparative analysis of discordant results for TN RIF and mWRD (GX RIF)**

| **TN RIF** | **TN RIF Quantity** | | | | | | **GX RIF** | **GX RIF Quantity** | | | | | |
| --- | --- | --- | --- | --- | --- | --- | --- | --- | --- | --- | --- | --- | --- |
|  | **High** | **Medium** | **Low** | **Very Low** | **Not recorded** | **Total** |  | **High** | **Medium** | **Low** | **Very Low** | **Not recorded** | **Total** |
| **Sensitive** | 3 | 0 | 0 | 3 | 1 | 7 | **Sensitive** | 1 | 9 | 58 | 27 | 8 | 103 |
| **Resistant** | 2 | 0 | 0 | 1 | 3 | 6 | **Resistant** | 0 | 1 | 12 | 1 | 4 | 18 |
| **Indeterminate** | 3 | 3 | 19 | 80 | 6 | 111 | **Indeterminate** | 0 | 0 | 0 | 3 | 0 | 3 |
| **Total** | 8 | 3 | 19 | 84 | 10 | 124 | **Total** | 1 | 10 | 70 | 31 | 12 | 124 |

**Table 3 (a) Concordance of TN& mWRD (with Clinical reference standard (Confirmed, Unconfirmed and Unlikely TB)**

|  | **Confirmed TB**  **(Clinical+**  **Microbiological Evidence)** | **Unconfirmed TB(Clinical/**  **Symptomatic Evidence)** | **Unlikely TB**  **(Neither microbiological nor clinical**  **/symptomatic evidence)** | **Total** |  | **Confirmed TB**  **(Clinical+**  **Microbiological Evidence)** | **Unconfirmed TB(Clinical/**  **Symptomatic Evidence)** | **Unlikely TB**  **(Neither microbiological nor clinical**  **/symptomatic evidence)** | Total |
| --- | --- | --- | --- | --- | --- | --- | --- | --- | --- |
| **TN** |  |  |  |  | GX |  |  |  |  |
| Detected | 485* | 1 | 0 | 486 | Detected | 429* | 0 | 0 | 486 |
| Not Detected | 212 | 1112 | 293 | 1617 | Not Detected | 268 | 1113 | 293 | 1617 |
| **Total** | 697 | 1113 | 293 | 2103 | Total | 697 | 1113 | 293 | 2103 |
| Sensitivity 69·6 (66, 73)  Specificity 99·9 (99·5, 100)  PPV 99·8 (98·9, 100)  NPV 84 (81·9, 85·9) | | | | | Sensitivity 61·5 (57·8, 65·2)  Specificity 100 (99·7, 100)  PPV 100 (99·1, 100)  NPV 80·6 (78·4, 82·6) | | | | |

*Total no. of samples detected both by TN and GX were 316, detailed analysis of extra samples picked up by TN&GX is given in Table 3(b) & 3(c) respectively

**Table 3(b) Comparative analysis of samples Positive by TN (negative by mWRD (GX)**

|  | **TN** | |  |
| --- | --- | --- | --- |
| **TN Quantity** | **Confirmed TB** | **Unconfirmed TB** | **Total** |
| **High** | 59 | 0 | 59 |
| **Low** | 28 | 0 | 28 |
| **Very low** | 69 | 0 | 69 |
| **Not recorded** | 13 | 1 | 14 |
| **Total** | 169 | 1 | 170* |

*76 out of 843 samples from AIIMS, New Delhi; 11 out of 348 samples from CMC, Vellore; and 83 out of 912 from HNH, Mumbai

**Table 3(c) Comparative analysis of samples Positive by mWRD (GX) (TN negative)**

|  | **GX** | |  |
| --- | --- | --- | --- |
| **GX Quantity** | **Confirmed TB** | **Unconfirmed TB** | **Total** |
| **High** | 1 | 0 | 1 |
| **Medium** | 2 | 0 | 2 |
| **Low** | 21 | 0 | 21 |
| **Very low** | 39 | 0 | 39 |
| **Not recorded** | 50 | 0 | 50 |
| **Total** | 113 | 0 | 113^ |

^50 out of 843 samples from AIIMS, New Delhi; 33 out of 348 samples from CMC, Vellore; and 30 out of 912 from HNH, Mumbai
